# Exchangeability of Measures of Association Before and After Exposure Status Is Flipped: Its Relationship With Confounding in the Counterfactual Model

**DOI:** 10.2188/jea.JE20210352

**Published:** 2023-08-05

**Authors:** Etsuji Suzuki, Michio Yamamoto, Eiji Yamamoto

**Affiliations:** 1 Department of Epidemiology, Graduate School of Medicine, Dentistry and Pharmaceutical Sciences, Okayama University, Okayama, Japan; 2 Graduate School of Environmental and Life Science, Okayama University, Okayama, Japan; 3 RIKEN Center for Advanced Intelligence Project, Tokyo, Japan; 4 Okayama University of Science, Okayama, Japan

**Keywords:** causality, causal inference, confounding, counterfactual, exchangeability, target population

## Abstract

**Background:**

The counterfactual definition of confounding is often explained in the context of exchangeability between the exposed and unexposed groups. One recent approach is to examine whether the measures of association (eg, associational risk difference) are exchangeable when exposure status is flipped in the population of interest. We discuss the meaning and utility of this approach, showing their relationships with the concept of confounding in the counterfactual framework.

**Methods:**

Three hypothetical cohort studies are used, in which the target population is the total population. After providing an overview of the notions of confounding in distribution and in measure, we discuss the approach from the perspective of exchangeability of measures of association (eg, *factual associational risk difference* vs *counterfactual associational risk difference*).

**Results:**

In general, if the measures of association are non-exchangeable when exposure status is flipped, confounding in distribution is always present, although confounding in measure may or may not be present. Even if the measures of association are exchangeable when exposure status is flipped, there could be confounding both in distribution and in measure. When we use risk difference or risk ratio as a measure of interest and the exposure prevalence in the population is 0.5, testing the exchangeability of measures of association is equivalent to testing the absence of confounding in the corresponding measures.

**Conclusion:**

The approach based on exchangeability of measures of association essentially does not provide a definition of confounding in the counterfactual framework. Subtly differing notions of confounding should be distinguished carefully.

## INTRODUCTION

Confounding is a major concern in estimating the causal effects of treatments or interventions. Since the publication of the seminal article by Greenland and Robins,^1^ the counterfactual approach to confounding has been widely accessible to epidemiologists, and the concept of confounding is now often explained within the counterfactual framework.^2^^–^^14^ Within this framework, researchers often compare ideal randomized controlled trials and observational studies in the context of the assumption of exchangeability between the exposed and unexposed groups.^9^^–^^13^ In sufficiently large randomized controlled trials with no loss to follow-up, confounding is not expected as long as investigators use an intention-to-treat analysis.^11^^,^^12^^,^^15^ In other more realistic settings, however, confounding is almost always expected to occur.

Multiple attempts have been made to explain the counterfactual definition of confounding in the field of epidemiology.^3^^,^^10^^,^^12^^,^^16^^,^^17^ One of these approaches is to examine whether the measures of association (such as associational risk differences and associational risk ratios) are exchangeable, or equivalent, when exposure status is “flipped” in the population of interest so that the exposed become the unexposed and vice versa.^18^ Basing the approach on *exchangeability of measures of association* may seem reasonable to many clinical researchers as an explanation of the definition of confounding because, in ideal randomized controlled trials, the exposed group and the unexposed group are expected to be comparable, or exchangeable, especially when the sample is large enough.^7^^,^^12^^,^^16^ From this perspective, some may infer that confounding occurs if and only if the measures of association are non-exchangeable when the exposure status is flipped.

In this article, we discuss the meaning and utility of this approach, showing their relationships with the concept of confounding in the counterfactual framework. In so doing, we carefully examine the relationships by distinguishing the subtly differing and hitherto underappreciated notions of confounding (ie, confounding in distribution and confounding in measure),^2^^,^^12^^,^^19^^,^^20^ for a better understanding of the concept of confounding. This paper assumes that readers have a basic knowledge of probabilities and statistics and focuses only on expected data without discussing random variation.

## NOTATIONS AND SETTINGS

As an illustration, we first consider a hypothetical cohort study in Table 1. We let *E* denote a binary exposure of interest (1 = exposed, 0 = unexposed) and *D* a binary outcome (1 = outcome occurred, 0 = outcome did not occur). For example, from the information of Study 1, we can readily calculate the associational risk difference as Pr(*D* = 1|*E* = 1) − Pr(*D* = 1|*E* = 0) = 360/600 − 160/400 = 1/5, and calculate the associational risk ratio as Pr(*D* = 1|*E* = 1)/Pr(*D* = 1|*E* = 0) = (360/600)/(160/400) = 3/2. The exposure prevalence in Study 1 is 0.6 (ie, Pr(*E* = 1) = 0.6). Recall that random error is ignored here, which is equivalent to viewing that the tabulated numbers in Table 1 represent summaries of a very large population with the trailing zeros removed. For simplicity, we assume that there is no loss to follow-up and that all variables are measured without error.

**Table 1.** Observed data in Study 1

	Exposed group (*E* = 1)	Unexposed group (*E* = 0)	Total population
Outcome occurred (*D* = 1)	360	160	520
Outcome did not occur (*D* = 0)	240	240	480
Total	600	400	1,000

In the counterfactual framework, we let *D^e^* denote the potential outcomes for an individual if, possibly contrary to fact, there had been interventions to set *E* to *e*. As a result, individuals can be classified into four response types: doomed, causal, preventive, and immune.^1^ In Table 2, we show underlying hypothetical data in terms of response types in Study 1, which is, though unobservable in the real world, integral to understanding the concept of confounding. Accordingly, in Table 3, we provide a hypothetical distribution of response types in Study 1, letting *p_i_*, *q_i_*, and *r_i_* (*i* = 1,…,4) be proportions of response type *i* in the exposed group, the unexposed group, and the total population, respectively. Under the assumption of consistency,^21^^,^^22^ the risk in the exposed group is given as Pr(*D* = 1|*E* = 1) = Pr(*D*^1^ = 1|*E* = 1) = *p*_1_ + *p*_2_ = 4/15 + 5/15 = 9/15. Similarly, the risk in the unexposed group is given as Pr(*D* = 1|*E* = 0) = Pr(*D*^0^ = 1|*E* = 0) = *q*_1_ + *q*_3_ = 4/10 + 0/10 = 4/10. Accordingly, as mentioned above, the associational risk difference is calculated as (*p*_1_ + *p*_2_) − (*q*_1_ + *q*_3_) = 9/15 − 4/10 = 1/5, while the associational risk ratio is calculated as (*p*_1_ + *p*_2_)/(*q*_1_ + *q*_3_) = (9/15)/(4/10) = 3/2.

**Table 2.** Underlying hypothetical data in terms of response types in Study 1

Response types	Potential outcomes		Exposed group (*E* = 1)	Unexposed group (*E* = 0)	Total population^a^

	*D* ^1^	*D* ^0^			
1 (doomed)	1	1	160	160	320
2 (causal)	1	0	200	40	240
3 (preventive)	0	1	40	0	40
4 (immune)	0	0	200	200	400
Total			600	400	1,000

^a^The number of those who had the outcome *D* in the total population is 520 in Table 1, which is obtained by summing the 160 exposed “doomed” individuals, the 200 exposed “causal” individuals, the 160 unexposed “doomed” individuals, and the 0 unexposed “preventive” individual. Likewise, the number of those did not have the outcome *D* in the total population is 480 in Table 1, which is obtained by summing the 40 exposed “preventive” individuals, the 200 exposed “immune” individuals, the 40 unexposed “causal” individuals, and the 200 unexposed “immune” individuals.

**Table 3.** Distributions of response types, measures of effect, and measures of association in Study 1

Response types	Potential outcomes		Proportion of types in		
	
	*D* ^1^	*D* ^0^	Exposed group (*E* = 1)	Unexposed group (*E* = 0)	Total population^a^
1 (doomed)	1	1	*p*_1_ = 4/15	*q*_1_ = 4/10	*r*_1_ = 8/25
2 (causal)	1	0	*p*_2_ = 5/15	*q*_2_ = 1/10	*r*_2_ = 6/25
3 (preventive)	0	1	*p*_3_ = 1/15	*q*_3_ = 0/10	*r*_3_ = 1/25
4 (immune)	0	0	*p*_4_ = 5/15	*q*_4_ = 5/10	*r*_4_ = 10/25

			Target population^b^		

			Exposed group (*E* = 1)	Unexposed group (*E* = 0)	Total population

Risk when exposed			*p*_1_ + *p*_2_ = **9/15**	*q*_1_ + *q*_2_ = 5/10	*r*_1_ + *r*_2_ = 14/25
Risk when unexposed			*p*_1_ + *p*_3_ = 5/15	*q*_1_ + *q*_3_ = **4/10**	*r*_1_ + *r*_3_ = 9/25
Measures of effect					
cRD			(*p*_1_ + *p*_2_) − (*p*_1_ + *p*_3_) = 4/15	(*q*_1_ + *q*_2_) − (*q*_1_ + *q*_3_) = 1/10	(*r*_1_ + *r*_2_) − (*r*_1_ + *r*_3_) = 1/5
cRR			(*p*_1_ + *p*_2_)/(*p*_1_ + *p*_3_) = 9/5	(*q*_1_ + *q*_2_)/(*q*_1_ + *q*_3_) = 5/4	(*r*_1_ + *r*_2_)/(*r*_1_ + *r*_3_) = 14/9

Measures of association in the “original” or factual situation					
Factual aRD			(*p*_1_ + *p*_2_) − (*q*_1_ + *q*_3_) = 9/15 − 4/10 = 1/5		
Factual aRR			(*p*_1_ + *p*_2_)/(*q*_1_ + *q*_3_) = (9/15)/(4/10) = 3/2		
Measures of association in the “flipped” or counterfactual situation					
Counterfactual aRD			(*q*_1_ + *q*_2_) − (*p*_1_ + *p*_3_) = 5/10 − 5/15 = 1/6		
Counterfactual aRR			(*q*_1_ + *q*_2_)/(*p*_1_ + *p*_3_) = (5/10)/(5/15) = 3/2		

aRD, associational risk difference; aRR, associational risk ratio; cRD, causal risk difference; cRR, causal risk ratio.

^a^The exposure prevalence of this cohort study is 0.6. Accordingly, *r_i_* can be calculated as *p_i_* × 0.6 + *q_i_* × 0.4.

^b^In this cohort study, only the numbers shown in bold can be observed under the assumption of consistency.^21^^,^^22^

## THE TARGET POPULATION PLAYS A KEY ROLE IN THE CONCEPT OF CONFOUNDING

The concept of target population is significant when explaining confounding because confounding depends on the population selected as the target of inference.^4^^,^^12^^,^^17^ Target parameters of causal inference, or measures of effect, cannot be defined unless the target population is clearly defined. For example, when the total population is used as the target population, the counterfactual risk when everyone in the total population is exposed is given in terms of response types as Pr(*D*^1^ = 1) = *r*_1_ + *r*_2_. In a similar manner, the counterfactual risk when everyone in the total population is unexposed is given as Pr(*D*^0^ = 1) = *r*_1_ + *r*_3_. Accordingly, in Table 3, causal risk difference in the total population is calculated as (*r*_1_ + *r*_2_) − (*r*_1_ + *r*_3_) = 14/25 − 9/25 = 1/5, whereas the causal risk ratio is calculated as (*r*_1_ + *r*_2_)/(*r*_1_ + *r*_3_) = (14/25)/(9/25) = 14/9. Once the information about these target parameters, or causal estimands, in the target population is available, the concept of confounding (or more strictly speaking, confounding in measure) becomes clearer by comparing the causal estimands with the corresponding measures of association.^2^^,^^12^^,^^19^^,^^20^

In the following discussion, the total population (ie, 0 < Pr(*E* = 1) < 1) is used as the target population because this is generally helpful in understanding the concept of confounding.^12^^,^^20^^,^^23^ However, the discussion is readily extendable to situations in which the target population is either the exposed group or the unexposed group, by setting Pr(*E* = 1) to equal 1 or 0, respectively. Note that we focus here on the three usual internal target populations.

## AN OVERVIEW OF TWO NOTIONS OF CONFOUNDING

In this section, we provide a brief overview of two subtly differing notions of confounding, ie, *confounding in distribution* and *confounding in measure*.^2^^,^^12^^,^^19^^,^^20^

First, we consider the notion of *confounding in distribution*, which makes reference to the distribution of potential outcomes. When using this notion, confounding is absent if and only if the groups that are actually exposed and unexposed are representative of what would have occurred had the total population been exposed and unexposed, respectively. Therefore, a necessary and sufficient condition for no confounding in distribution is generally given by

$$\begin{array}{rl} & \{(r_{1}+r_{2})=(p_{1}+p_{2})\}\wedge \{(r_{1}+r_{3})=(q_{1}+q_{3})\}\\ & \Leftrightarrow \{(p_{1}+p_{2})=(q_{1}+q_{2})\}\wedge \{(p_{1}+p_{3})=(q_{1}+q_{3})\}, \end{array}$$ (1)

which is violated in Table 3. Note that *r_i_* can be calculated as *p_i_* × Pr(*E* = 1) + *q_i_* × Pr(*E* = 0). Equation 1 is equivalent to *D^e^* ⫫ *E* (*e* = 0, 1), which is often termed an *exchangeability* condition in the counterfactual model.^1^^,^^13^

In many cases, confounding is also defined with respect to a specific effect measure (ie, *confounding in measure*), and confounding is scale-dependent; it may be absent on one scale (eg, the risk difference scale) but present on another (eg, the risk ratio scale). If we use risk difference as a measure of interest, confounding is absent if and only if causal risk difference is identical to associational risk difference. Thus, a necessary and sufficient condition for no confounding in measure for the risk difference is given by

$$\begin{array}{rl} & (r_{1}+r_{2})-(r_{1}+r_{3})=(p_{1}+p_{2})-(q_{1}+q_{3})\\ & \Leftrightarrow (p_{2}-p_{3})\times \mathrm{Pr}(E=1)+(q_{2}-q_{3})\times \mathrm{Pr}(E=0)\\ & \;=(p_{1}+p_{2})-(q_{1}+q_{3})\\ & \Leftrightarrow (p_{1}+p_{3})\times \mathrm{Pr}(E=1)+(p_{1}+p_{2})\times \mathrm{Pr}(E=0)\\ & \;=(q_{1}+q_{3})\times \mathrm{Pr}(E=1)+(q_{1}+q_{2})\times \mathrm{Pr}(E=0), \end{array}$$ (2)

which is met in Table 3. Meanwhile, if we use risk ratio as a measure of interest, confounding is absent if and only if causal risk ratio is identical to associational risk ratio. Thus, a necessary and sufficient condition for no confounding in measure for the risk ratio is given by

$$\begin{array}{rl} & \frac{r_{1}+r_{2}}{r_{1}+r_{3}}=\frac{p_{1}+p_{2}}{q_{1}+q_{3}}\\ & \Leftrightarrow \frac{\left(p_{1}+p_{2})\times \mathrm{Pr}(E=1)+(q_{1}+q_{2})\times \mathrm{Pr}(E=0\right)}{\left(p_{1}+p_{3})\times \mathrm{Pr}(E=1)+(q_{1}+q_{3})\times \mathrm{Pr}(E=0\right)}\\ & \;=\frac{p_{1}+p_{2}}{q_{1}+q_{3}}\\ & \Leftrightarrow (p_{1}+p_{2})\{(p_{1}+p_{3})-(q_{1}+q_{3})\}\times \mathrm{Pr}(E=1)\\ & \;=(q_{1}+q_{3})\{(q_{1}+q_{2})-(p_{1}+p_{2})\}\times \mathrm{Pr}(E=0), \end{array}$$ (3)

which is violated in Table 3. Obviously, risk ratios in equation 3 are defined when the denominators are not 0. Note that, if equation 1 holds, equations 2 and 3 always hold. That is, equation 1 is stronger than equations 2 and 3. As clearly seen in the last lines of equations 2 and 3, when the total population is used as the target population, confounding in measure depends on the exposure prevalence in the population.

To summarize, when the total population is used as the target population, no confounding in distribution is stronger than no confounding in measure.^2^^,^^12^^,^^19^^,^^20^ Accordingly, as illustrated in Table 3, even when confounding in distribution is present, confounding in measure is not always present. Furthermore, while confounding in distribution is scale-independent, confounding in measure is scale-dependent. In Table 3, confounding in measure for the risk difference is absent, whereas confounding in measure for the risk ratio is present. This example illustrates that it is crucial to distinguish between the two notions of confounding. A related discussion about the odds ratio is available elsewhere.^20^ When either the exposed or unexposed group is used as the target population, distinguishing between the two notions becomes a subtle issue because the necessary and sufficient conditions for no confounding become equivalent. This has been discussed in greater detail in the literature.^2^^,^^12^^,^^19^^,^^20^

The next section discusses the approach from the perspective of exchangeability of measures of association.

## EXCHANGEABILITY OF MEASURES OF ASSOCIATION

To examine whether the measures of association are exchangeable when exposure status is flipped, we need to consider two unobservable quantities: what would have occurred in the actual exposed group had the subjects not been exposed, and what would have occurred in the actual unexposed group had the subjects been exposed. In the counterfactual framework, the former quantity in Table 3 is described in terms of response types as Pr(*D*^0^ = 1|*E* = 1) = *p*_1_ + *p*_3_ = 4/15 + 1/15 = 5/15. Similarly, the latter quantity is described as Pr(*D*^1^ = 1|*E* = 0) = *q*_1_ + *q*_2_ = 4/10 + 1/10 = 5/10. Accordingly, in Study 1, the associational risk difference when the exposure status is flipped is calculated as (*q*_1_ + *q*_2_) − (*p*_1_ + *p*_3_) = 5/10 − 5/15 = 1/6. We refer to this measure as *counterfactual associational risk difference*. Likewise, the associational risk ratio when the exposure status is flipped is calculated as (*q*_1_ + *q*_2_)/(*p*_1_ + *p*_3_) = (5/10)/(5/15) = 3/2. We refer to this measure as *counterfactual associational risk ratio*.

First, let us consider a situation in which we use risk difference as a measure of interest. As mentioned above, associational risk difference in the “original” or factual situation is described as (*p*_1_ + *p*_2_) − (*q*_1_ + *q*_3_), which we refer here to as *factual associational risk difference*. Then, factual associational risk difference is identical to counterfactual associational risk difference if and only if the following equation holds:

$$\begin{array}{rl} & (p_{1}+p_{2})-(q_{1}+q_{3})=(q_{1}+q_{2})-(p_{1}+p_{3})\\ & \Leftrightarrow \{(q_{1}+q_{2})=(p_{1}+p_{2})+\alpha \}\wedge \{(p_{1}+p_{3})=(q_{1}+q_{3})+\alpha \}, \end{array}$$ (4)

for some scalar *α* bounded between −1 and 1. Equation 4 is weaker than equation 1 (ie, no confounding in distribution) and is neither stronger nor weaker than equation 2 (ie, no confounding in measure for the risk difference). When *α* = 0, equation 4 becomes equivalent to equation 1. Although equation 2 holds in Table 3, which means that confounding in measure for the risk difference is absent, equation 4 does not hold.

Next, let us consider a situation in which we use risk ratio as a measure of interest. As mentioned above, associational risk ratio in the “original” or factual situation is described as (*p*_1_ + *p*_2_)/(*q*_1_ + *q*_3_), which we refer here to as *factual associational risk ratio*. Then, factual associational risk ratio is identical to counterfactual associational risk ratio if and only if the following equation holds:

$$\begin{array}{rl} & \frac{p_{1}+p_{2}}{q_{1}+q_{3}}=\frac{q_{1}+q_{2}}{p_{1}+p_{3}}\\ & \Leftrightarrow \{(q_{1}+q_{2})=(p_{1}+p_{2})\beta \}\wedge \{(p_{1}+p_{3})=(q_{1}+q_{3})\beta \}, \end{array}$$ (5)

for some positive scalar *β* (see eMaterial 1 for the proof). Obviously, risk ratios in equation 5 are defined when the denominators are not 0. Equation 5 is weaker than equation 1 (ie, no confounding in distribution) and is neither stronger nor weaker than equation 3 (ie, no confounding in measure for the risk ratio). When *β* = 1, equation 5 becomes equivalent to equation 1. Although equation 3 does not hold in Table 3, which means that confounding in measure for the risk ratio is present, equation 5 holds, where *β* = 5/6. Thus, in Table 3, the patterns of confounding in measure and exchangeability of measures of association are, so to speak, reversed by the measures of interest; factual associational risk difference is equivalent not to counterfactual associational risk difference but to causal risk difference, whereas factual associational risk ratio is equivalent not to causal risk ratio but to counterfactual associational risk ratio.

In general, even if the measures of association are exchangeable before and after exposure status is flipped, there could be confounding both in distribution and in measure. When the exposure prevalence in the population is 0.5, however, equations 2 and 3 become equivalent to equations 4 and 5, respectively. Thus, when we use risk difference or risk ratio as a measure of interest and the exposure prevalence is 0.5, testing the exchangeability of measures of association before and after exposure status is flipped is equivalent to testing the absence of confounding in the corresponding measures. This may provide an interpretation, or rationale, of testing exchangeability of measures of association in such specific situations, although it does not hold for odds ratio. As an illustrative example, Study 2 shows what would happen to the data in Study 1 if the exposure prevalence was 0.5 (eTable 1 and eTable 2 in eMaterial 2). In eTable 2, causal risk difference, factual associational risk difference, and counterfactual associational risk difference are 11/60, 1/5, and 1/6, respectively; neither equations 2 nor 4 hold. Meanwhile, causal risk ratio, factual associational risk ratio, and counterfactual associational risk ratio are all 3/2; both equations 3 and 5 hold, where *β* = 5/6 in equation 5.

The presence of confounding in measure could be a marker of the presence of confounding in distribution. Some may wonder whether non-exchangeability between factual measures of association and counterfactual measures of association (for either risk difference or risk ratio) could be used as another marker of the presence of confounding in distribution because the non-exchangeable measures of association are a sufficient condition for confounding in distribution (ie, violation of equation 1). Recall, however, that the non-exchangeable measures of association are neither a necessary nor a sufficient condition for confounding in measure. In other words, unlike confounding in measure, the targets of the approach based on exchangeability of measures of association are not measures of effect, or target parameters of causal inference. Thus, from the counterfactual definitions of confounding, the utility of examining the non-exchangeable measures of association may be generally limited. Irrespective of whether measures of association are exchangeable or not, researchers need to consider controlling for confounding in either distribution or measure from theoretical and analytical perspectives.

Finally, it is worth mentioning that, even when confounding in distribution is present (that is, equation 1 does not hold), equations 4 and 5 can simultaneously hold if {(*p*_1_ + *p*_2_) = (*q*_1_ + *q*_3_)} ∧ {(*p*_1_ + *p*_3_) = (*q*_1_ + *q*_2_)}, irrespective of the exposure prevalence. In this case, both factual and counterfactual measures of association trivially take null values. As an illustrative example, see Study 3 (eTable 3 and eTable 4 in eMaterial 2), where the numbers of unexposed subjects with *D* = 1 and *D* = 0 are flipped from those of Study 1. In Study 3, *α* = −4/15 in equation 4, while *β* = 5/9 in equation 5. Table 4 summarizes the three hypothetical examples.

**Table 4.** Summary of the three hypothetical cohort studies when the target population is the total population

Condition^a^	Study 1^b^	Study 2^c^	Study 3^d^
No confounding in distribution (Eq. 1)^e^	False	False	False
No confounding in measure			
No confounding in risk difference (Eq. 2)	True	False	False
No confounding in risk ratio (Eq. 3)	False	True	False
Exchangeability of measures of association			
Exchangeability of associational risk difference (Eq. 4)	False	False	True
Exchangeability of associational risk ratio (Eq. 5)	True	True	True

^a^Equation 1 is generally stronger than equations 2, 3, 4, and 5. Under the assumption of (*p*_2_ = *q*_2_) ∧ (*p*_3_ = *q*_3_), however, equations 1, 2, 3, 4, and 5 all become identical to the exchangeability of “background risks” (ie, *p*_1_ = *q*_1_). See equations S1, S2, S3, S4, and S5 in eMaterial 3, respectively.

^b^See Table 1, Table 2, and Table 3.

^c^See eTable 1 and eTable 2 in eMaterial 2.

^d^See eTable 3 and eTable 4 in eMaterial 2.

^e^Equation 1 is equivalent to *D^e^* ⫫ *E* (*e* = 0, 1), which is often termed an exchangeability condition in the counterfactual model. See text for details.

On a related issue, when discussing the concept of confounding, a recent study focused on the exchangeability of “background risks,” explaining that the background risks are “not caused by, and thus independent of, the exposure of interest.”^18^^ (p. 95)^ In general, however, the exchangeability of background risks is not a primary issue of the concept of confounding. See eMaterial 3 for further discussion.

## CONCLUSION

From the perspective of counterfactual reasoning, we discussed the meaning and utility of examining whether the measures of association are exchangeable when exposure status is flipped in the population of interest. In so doing, we used the total population as the target population, which clarifies the significance of distinguishing the subtly differing notions of confounding in distribution and confounding in measure.^12^^,^^20^^,^^23^ Distinguishing these notions is important for understanding the meaning and utility of the approach based on exchangeability of measures of association. In general, if the measures of association are non-exchangeable when exposure status is flipped, confounding in distribution is always present, although confounding in measure may or may not be present. In essence, however, the approach based on exchangeability of measures of association does not provide a definition of confounding in the counterfactual framework. In addition, the exchangeability of the so-called background risks is neither a necessary nor a sufficient condition for no confounding, irrespective of the notions of confounding.

Despite its significance, there is some confusion in the concept of confounding,^4^^,^^12^ and the notions of confounding in distribution and confounding in measure have been relatively underappreciated.^6^^,^^24^^,^^25^ As examples of the confusion surrounding the concept of confounding, a review article by Gatto et al explains the conditions for no confounding that are implicitly derived from the notion of confounding in distribution,^24^ whereas Maldonado implicitly used the notion of confounding in measure when discussing the concept of confounding.^25^ In addition to these notions of confounding, however, a further distinction can be drawn between *confounding in expectation* and *realized confounding*.^7^^,^^12^^,^^19^ As mentioned at the end of the Introduction section, this paper focuses only on expected data without discussing random variation, and thus the distinction between these two notions of confounding is irrelevant. Nonetheless, distinguishing these notions is important for a clearer understanding of the concept of bias.^11^^,^^26^ A typology of the four notions of confounding is available elsewhere.^12^ Our study further highlights that subtly differing notions of confounding should be distinguished carefully.

## SUPPLEMENTARY MATERIAL

eMaterial 1: Proof of equation 5

eMaterial 2: Studies 2 and 3

eMaterial 3: Exchangeability of “background risks”

eTable 1. Observed data in Study 2

eTable 2. Distributions of response types, measures of effect, and measures of association in Study 2

eTable 3. Observed data in Study 3

eTable 4. Distributions of response types, measures of effect, and measures of association in Study 3
