# Supplementary material for: Exchangeability of Measures of Association Before and After Exposure Status Is Flipped: Its Relationship With Confounding in the Counterfactual Model
Source: J Epidemiol. 2023 Aug 5;33(8):385–9. doi: 10.2188/jea.JE20210352 (PMC10319525; doi:10.2188/jea.JE20210352)
Supplement: Supplementary file 1 [file je-33-385-s001.pdf]

Exchangeability of Measures of Association Before and After Exposure Status Is Flipped: Its Relationship With Confounding in the Counterfactual Model

eMaterial 1: Proof of equation 5

We set $K = p_1 + p_2$, $L = q_1 + q_3$, $M = q_1 + q_2$, and $N = p_1 + p_3$, where $K \geq 0$, $L > 0$, $M \geq 0$, and $N > 0$. Then, equation 5 is proved as follows:

$$\frac{K}{L} = \frac{M}{N}$$
$$\Leftrightarrow \frac{K}{L} = \frac{M}{N} \wedge (K \neq 0 \vee K = 0)$$
$$\Leftrightarrow \left(\frac{K}{L} = \frac{M}{N} \wedge K \neq 0\right) \vee \left(\frac{K}{L} = \frac{M}{N} \wedge K = 0\right)$$
$$\Leftrightarrow \left(\frac{M}{K} = \frac{N}{L} \wedge K \neq 0\right) \vee (M = 0 \wedge K = 0)$$
$$\Leftrightarrow \{(\exists\beta > 0\ (M = K\beta \wedge N = L\beta)) \wedge K \neq 0\} \vee \{(\exists\beta > 0\ (M = K\beta \wedge N = L\beta)) \wedge K = 0\}$$
$$\Leftrightarrow (\exists\beta > 0\ (M = K\beta \wedge N = L\beta)) \wedge (K \neq 0 \vee K = 0)$$
$$\Leftrightarrow \exists\beta > 0\ (M = K\beta \wedge N = L\beta) \quad \blacksquare$$

eMaterial 2: Studies 2 and 3

See eTables 1 and 2 for Study 2, and eTables 3 and 4 for Study 3.

eMaterial 3: Exchangeability of "background risks"

When discussing the concept of confounding, a recent study focused on the exchangeability of "background risks," explaining that the background risks are "not caused by, and thus independent of, the exposure of interest."[1 (p. 95)] From a perspective of the counterfactual model, the background risks represent the proportions of the "doomed" individuals in the exposed and unexposed groups (i.e., p_1 and q_1, respectively, in Table 3 and eTables 2 and 4). As clearly seen in equations 1–3, however, the exchangeability of background risks (i.e., $p_1 = q_1$) is neither a necessary nor a sufficient condition for no confounding, irrespective of the notions of confounding. Furthermore, as shown in equations 4 and 5, the exchangeability of background risks is neither a necessary nor a sufficient condition for exchangeability of measures of association. Under a certain strong assumption, however, the exchangeability of background risks is important, as discussed below.

Let us assume that the proportions of the "causal" individuals and "preventive" individuals are

respectively identical between the exposed and unexposed groups (i.e., $(p_2 = q_2) \wedge (p_3 = q_3)$). Under this assumption, equation 1 regarding no confounding in distribution becomes

$$\{(p_1 + p_2) = (q_1 + p_2)\} \wedge \{(p_1 + p_3) = (q_1 + p_3)\}$$
$$\Leftrightarrow p_1 = q_1, \qquad \text{(Eq. S1)}$$

which shows that the exchangeability of background risks is a necessary and sufficient condition for no confounding in distribution. Note that, when background risks are exchangeable under this assumption, the distributions of the four response types are fully identical between the exposed and unexposed groups, i.e., $(p_1, p_2, p_3, p_4) = (q_1, q_2, q_3, q_4) \Leftrightarrow \{D^1, D^0\} \perp\!\!\!\perp E$. This is often termed a *full exchangeability* condition,[2] which is stronger than the exchangeability condition mentioned in the main text.

Likewise, under the assumption of $(p_2 = q_2) \wedge (p_3 = q_3)$, equation 2 regarding no confounding in measure for the risk difference becomes

$$(p_1 + p_3) \times \Pr(E = 1) + (p_1 + p_2) \times \Pr(E = 0) = (q_1 + p_3) \times \Pr(E = 1) + (q_1 + p_2) \times \Pr(E = 0)$$
$$\Leftrightarrow p_1 = q_1, \qquad \text{(Eq. S2)}$$

and equation 3 regarding no confounding in measure for the risk ratio becomes

$$(p_1 + p_2)(p_1 - q_1) \times \Pr(E = 1) = (q_1 + q_3)(q_1 - p_1) \times \Pr(E = 0)$$
$$\Leftrightarrow (p_1 - q_1)\{(p_1 + p_2) \times \Pr(E = 1) + (q_1 + q_3) \times \Pr(E = 0)\} = 0$$
$$\Leftrightarrow p_1 = q_1. \quad \text{(Eq. S3)}$$

Note that, as long as the exposure prevalence is greater than 0 and smaller than 1, the formula in the curly brackets in the second line of equation S3 could take 0 if and only if $(p_1, p_2, p_3, p_4) = (q_1, q_2, q_3, q_4) = (0,0,0,1)$, which is *a priori* excluded because denominators of the risk ratios in equation 3 become 0. Equations S2 and S3 show that, under the assumption of $(p_2 = q_2) \wedge (p_3 = q_3)$, the exchangeability of background risks is a necessary and sufficient condition for no confounding in measure, irrespective of the exposure prevalence in the total population. Thus, under the assumption of $(p_2 = q_2) \wedge (p_3 = q_3)$, the distinction between the two subtly differing notions of confounding in distribution and in measure becomes less clear, even if the target is the total population.

Furthermore, under the assumption of $(p_2 = q_2) \wedge (p_3 = q_3)$, the exchangeability of background risks occurs if and only if the measures of association are exchangeable when the exposure status is flipped. Under this assumption, the first line of equation 4 becomes

$$(p_1 + p_2) - (q_1 + p_3) = (q_1 + p_2) - (p_1 + p_3)$$
$$\Leftrightarrow 2(p_1 - q_1) = 0$$
$$\Leftrightarrow p_1 = q_1, \qquad \text{(Eq. S4)}$$

and the first line of equation 5 becomes

$$\frac{p_1 + p_2}{q_1 + p_3} = \frac{q_1 + p_2}{p_1 + p_3}$$
$$\Leftrightarrow (p_1 + p_2)(p_1 + p_3) = (q_1 + p_2)(q_1 + p_3)$$
$$\Leftrightarrow (p_1^2 - q_1^2) + p_3(p_1 - q_1) + p_2(p_1 - q_1) = 0$$
$$\Leftrightarrow (p_1 - q_1)\{(p_1 + q_1) + p_3 + p_2\} = 0$$
$$\Leftrightarrow p_1 = q_1. \qquad \text{(Eq. S5)}$$

Note that the formula in the curly brackets in the fourth line of equation S5 could take 0 if and only if $(p_1, p_2, p_3, p_4) = (q_1, q_2, q_3, q_4) = (0,0,0,1)$, which is *a priori* excluded because denominators of the risk ratios become 0. Equations S4 and S5 show that, under the assumption of $(p_2 = q_2) \wedge (p_3 = q_3)$, the exchangeability of background risks becomes a necessary and sufficient condition for exchangeability of measures of association.

To summarize, in the discussion of confounding, the role of background risks is restricted to situations in which one can make a strong assumption; when the proportions of the "causal" individuals and "preventive" individuals are assumed to be respectively identical between the exposed and unexposed groups (i.e., $(p_2 = q_2) \wedge (p_3 = q_3)$), necessary and sufficient conditions for no confounding in distribution (Eq. S1), no confounding in measure (Eqs. S2 and S3), and exchangeability of measures of association (Eqs. S4 and S5) are all identical, that is the exchangeability of background risks (i.e., $p_1 = q_1$). In general, however, the exchangeability of background risks is not a primary issue of the concept of confounding.

References

1. Bours MJL. A nontechnical explanation of the counterfactual definition of confounding. J Clin Epidemiol. 2020;121:91–100.
2. Hernán MA, Robins JM. Causal Inference: What If. Boca Raton, FL. Chapman & Hall/CRC; 2020.
3. Cole SR, Frangakis CE. The consistency statement in causal inference: a definition or an assumption? Epidemiology. 2009;20:3–5.
4. VanderWeele TJ. Concerning the consistency assumption in causal inference. Epidemiology. 2009;20:880–883.

eTable 1. Observed data in Study 2

	Exposed group ($E = 1$)	Unexposed group ($E = 0$)	Total population
Outcome occurred ($D = 1$)	300	200	500
Outcome did not occur ($D = 0$)	200	300	500
Total	500	500	1,000

eTable 2. Distributions of response types, measures of effect, and measures of association in Study 2

Response types	Potential outcomes		Proportion of types in		
	D^1	D^0	Exposed group ($E = 1$)	Unexposed group ($E = 0$)	Total population[a]
1 (doomed)	1	1	$p_1 = 4/15$	$q_1 = 4/10$	$r_1 = 20/60$
2 (causal)	1	0	$p_2 = 5/15$	$q_2 = 1/10$	$r_2 = 13/60$
3 (preventive)	0	1	$p_3 = 1/15$	$q_3 = 0/10$	$r_3 = 2/60$
4 (immune)	0	0	$p_4 = 5/15$	$q_4 = 5/10$	$r_4 = 25/60$
			Target population[b]		
			Exposed group ($E = 1$)	Unexposed group ($E = 0$)	Total population
Risk when exposed			$p_1 + p_2 = \mathbf{9/15}$	$q_1 + q_2 = 5/10$	$r_1 + r_2 = 33/60$
Risk when unexposed			$p_1 + p_3 = 5/15$	$q_1 + q_3 = \mathbf{4/10}$	$r_1 + r_3 = 22/60$
Measures of effect					
cRD			$(p_1 + p_2) - (p_1 + p_3) = 4/15$	$(q_1 + q_2) - (q_1 + q_3) = 1/10$	$(r_1 + r_2) - (r_1 + r_3) = 11/60$
cRR			$(p_1 + p_2)/(p_1 + p_3) = 9/5$	$(q_1 + q_2)/(q_1 + q_3) = 5/4$	$(r_1 + r_2)/(r_1 + r_3) = 3/2$
Measures of association in the “original” or factual situation					
Factual aRD			$(p_1 + p_2) - (q_1 + q_3) = 9/15 - 4/10 = 1/5$		
Factual aRR			$(p_1 + p_2)/(q_1 + q_3) = (9/15)/(4/10) = 3/2$		
Measures of association in the “flipped” or counterfactual situation					
Counterfactual aRD			$(q_1 + q_2) - (p_1 + p_3) = 5/10 - 5/15 = 1/6$		
Counterfactual aRR			$(q_1 + q_2)/(p_1 + p_3) = (5/10)/(5/15) = 3/2$		

aRD, associational risk difference; aRR, associational risk ratio; cRD, causal risk difference; cRR, causal risk ratio.

[a]The exposure prevalence of this cohort study is 0.5. Accordingly, r_i can be calculated as $p_i \times 0.5 + q_i \times 0.5$.

[b]In this cohort study, only the numbers shown in bold can be observed under the assumption of consistency.[3, 4]

eTable 3. Observed data in Study 3

	Exposed group ($E = 1$)	Unexposed group ($E = 0$)	Total population
Outcome occurred ($D = 1$)	360	240	600
Outcome did not occur ($D = 0$)	240	160	400
Total	600	400	1,000

eTable 4. Distributions of response types, measures of effect, and measures of association in Study 3

Response types	Potential outcomes		Proportion of types in		
	D^1	D^0	Exposed group ($E = 1$)	Unexposed group ($E = 0$)	Total population[a]
1 (doomed)	1	1	$p_1 = 4/15$	$q_1 = 3/15$	$r_1 = 18/75$
2 (causal)	1	0	$p_2 = 5/15$	$q_2 = 2/15$	$r_2 = 19/75$
3 (preventive)	0	1	$p_3 = 1/15$	$q_3 = 6/15$	$r_3 = 15/75$
4 (immune)	0	0	$p_4 = 5/15$	$q_4 = 4/15$	$r_4 = 23/75$
			Target population[b]		
			Exposed group ($E = 1$)	Unexposed group ($E = 0$)	Total population
Risk when exposed			$p_1 + p_2 = \mathbf{9/15}$	$q_1 + q_2 = 5/15$	$r_1 + r_2 = 37/75$
Risk when unexposed			$p_1 + p_3 = 5/15$	$q_1 + q_3 = \mathbf{9/15}$	$r_1 + r_3 = 33/75$
Measures of effect					
cRD			$(p_1 + p_2) - (p_1 + p_3) = 4/15$	$(q_1 + q_2) - (q_1 + q_3) = -4/15$	$(r_1 + r_2) - (r_1 + r_3) = 4/75$
cRR			$(p_1 + p_2)/(p_1 + p_3) = 9/5$	$(q_1 + q_2)/(q_1 + q_3) = 5/9$	$(r_1 + r_2)/(r_1 + r_3) = 37/33$
Measures of association in the “original” or factual situation					
Factual aRD			$(p_1 + p_2) - (q_1 + q_3) = 9/15 - 9/15 = 0$		
Factual aRR			$(p_1 + p_2)/(q_1 + q_3) = (9/15)/(9/15) = 1$		
Measures of association in the “flipped” or counterfactual situation					
Counterfactual aRD			$(q_1 + q_2) - (p_1 + p_3) = 5/15 - 5/15 = 0$		
Counterfactual aRR			$(q_1 + q_2)/(p_1 + p_3) = (5/15)/(5/15) = 1$		

aRD, associational risk difference; aRR, associational risk ratio; cRD, causal risk difference; cRR, causal risk ratio.

[a]The exposure prevalence of this cohort study is 0.6. Accordingly, r_i can be calculated as $p_i \times 0.6 + q_i \times 0.4$.

[b]In this cohort study, only the numbers shown in bold can be observed under the assumption of consistency.[3, 4]
